# Medical History from the Earliest Times

**Published:** 1893-07-22

**Authors:** E. T. Withington


### Medical History from the Earliest Times.

By E. T. Withington, M.A., M.B. Oxon.

### LXIII.—THE BRUNONIAN SYSTEM.

Everyone, it has been said, is born a disciple of Plato or of Aristotle, with a deductive or an inductive mental tendency. The former class is impatient of details, eager to establish general principles, given rather to reflection than to observation, and prefers philosophy to the physical sciences. The inductive mind, on the contrary, relies less on intuition than upon the study of nature, devotes itself to the investigation of facts before attempting to explain them, and prefers to suspend its judgment rather than risk a hasty generalisation. These two tendencies, the scientific and the systematic, may be traced through the history of medicine, and stand out in striking contrast at the close of last century. On the one side pathologists and clinicians were establishing the basis for that progressive investigation which has revolutionised the healing art, while on the other attempts were made to give immediate explanations of life and disease, and to lay down universal laws of treatment in the ingenious "systems" of Brown and Hahnemann.

Life, according to Brown, is a forced state produced by the action of stimuli on the "excitability" of the body. The stimuli are external and internal, warmth, food, the fluids of the body, muscular motion, the senses, passions, &c. Excitability is a mysterious something possessed by all living beings, which is continually being used up and renewed during life, and which varies inversely with the stimulus. To take an illustration given by one of his pupils. Suppose a furnace continually supplied with coal which will only burn so long as it is blown. Then the fire will represent life, the coal excitability, and the bellows the stimulus. The fire may be put out in two ways, by ceasing to blow, or by blowing so hard that the coal is consumed more rapidly than it is supplied. Similarly death occurs, either when the stimulus sinks below a certain level, or when it is so violent as to exhaust the excitability; and, on the other hand, life is most stable and vigorous when both stimulus and excitability are of medium amount.

Brown's pathology is exactly parallel with his physiology. Diseases are due either to excessive or deficient excitement (*i.e.*, life), and are termed sthenic or asthenic, according as they arise from the former or latter cause. Asthenic diseases are far the most common, forming 97 per cent. of the whole, and they may be produced either by deficient stimulus (direct debility), or deficient excitability, following excessive stimulus (indirect debility). This may be made clearer by an illustration given by Brown himself, and, according to his biographers, frequently exemplified by him. A man slightly below par, or with a tendency to direct debility, drinks five glasses of wine. This, by taking off the excess of excitability, and adding to the stimulus, brings him to the normal state of health. He then drinks five more glasses, and his original state is now reversed, the stimulus being in excess and the excitability deficient. But, says the jovial Brown, we are not so flimsily made as not to stand a little excess, and the man is still in perfect health, though with a tendency to sthenic disease, which is increased on his taking five further glasses. Finally, should he drink 20 (!) glasses of wine the excitability becomes exhausted, the excitement sinks with it, and the man falls into a comatose state from indirect debility. He remains thus for some hours, and, the stimulus being reduced to its lowest point, excitability rapidly reaccumulates, so that on awaking he finds himself in the opposite condition of direct debility, with headache, shaky hands, &c., symptoms which may be readily removed by taking a slight stimulant to get rid of the excess of excitability. It follows from this pathology that diseases differ from one another and from the state of health, not in nature, but only in degree, and Brown declares that "the huge volumes on diagnosis" of the "old school" are now unnecessary, for when a physician is called to a case he has merely to decide three questions—Is the disease general or local? Is it sthenic or asthenic? What is its degree?

If the above theory be true, the rule of treatment is as simple as it is obvious—we must restore the normal degree of excitement by regulating the stimulus. According to Brown, all modes of medical treatment are forms of stimulus, differing only in degree, and he arranges the chief of them in the following order of relative efficacy: Opium, camphor, ammonia, musk, alcohol, active exercise, stimulant food, warmth, passive exercise, low diet, cold, purgatives, bleeding. The last five of these may conveniently be termed debilitants, since the excitement they produce is below the normal, and it is by them that the comparatively rare sthenic diseases, including small-pox, measles, and pneumonia must be treated. Thus when Brown's son, aged six, had measles, he stript him half naked, reduced his diet to "fluid vegetable matter," and let him run about as he pleased, whereupon he rapidly recovered. Opium is the greatest of stimulants, and is the sheet-anchor in treating all diseases of debility direct or indirect. In the former the excessive excitability should be removed by small doses gradually increased, while cases of indirect debility must be met by a stimulus slightly less than that which has produced the exhaustion. To use Brown's own words, "In direct debility, where the redundancy of excitability does not for a time admit of much stimulus, ten or twelve drops of laudanum should be given every quarter of an hour till the patient sleeps. After sleep, when some of the excessive excitability is worn off, a double quantity should be added, and gradually increased till the healthy state is regained. In indirect debility 150 drops (!) should be forthwith thrown in, and the superadditions made less and less." Local diseases are comparatively rare; they are characterised by the absence of any premonitory stage, which is always present in general affections, and they are to be treated on the same principles.

Such are the essential points of what is probably the simplest, most original, and most philosophic system of medicine ever invented. Compared with it the ancient methodism and the later homœopathy are little more than rules of treatment, and if it be the object of medicine to discover a philosophic theory on which may be based a simple and universal law of practice, then John Brown must be placed above Hippocrates. His definition of life shows striking resemblances to the views now accepted, and was compared at the time with the doctrine of John Locke; for just as that great medical philosopher declared that the mind contains no ideas in itself, but merely the power of receiving and developing them, so Brown held that the body has no innate life, but only a capacity for developing it from the action of stimuli. So, too, his theory that diseases are abnormal vital processes agrees with that now adopted, and which had already been advanced by Boerhaave in his famous definition, "Morbus est vita praeter naturam." Brown further did good service by accentuating the reactive power of the body, by opposing excessive depletion, and by showing that inflammation might be a sign rather of deficient than of excessive vital action. Finally, his assertion that life and death, sickness and health, the causation and cure of disease, the decay and renewal of nature, are all different manifestations of one and the same activity, shows that, like Paracelsus, with whom he is frequently compared, Brown was by no means destitute of genius.

But being based upon reflection rather than observation, Brunonianism failed in practice, and it has been considered here chiefly because it shows in a most striking way the defects of the systematic method. To say with some historians that the new doctrine cost as many lives as did the wars of Napoleon would indeed be an exaggeration, but the examples of treatment given above show that to accomplish this feat it only required to be sufficiently widely accepted. Happily it was not widely accepted. All systems of medicine tend to close the way to further progress, the more so the greater their completeness and finality, and Brown's system was especially antagonistic to the forms of medical progress then inaugurated, the local pathology of Morgagni, and the local diagnosis of Auenbrugger and Laennec. Moreover, those physicians who must have a "system" at any cost, were offered an attractive alternative in the doctrine of Hahnemann, which, though inferior in philosophic completeness and originality to that of Brown, possessed an immense advantage in the safety with which it might be applied in practice.

